# Knockdown of *FAS2* Impairs Fecundity by Inhibiting Lipid Accumulation and Increasing Glycogen Storage in *Locusta migratoria*

**DOI:** 10.3390/insects16020120

**Published:** 2025-01-26

**Authors:** Jiaying Xu, Ya Tang, Yi Jin, Tingting Ma, Chen Zhang, Jianan Lou, Bin Tang, Shigui Wang

**Affiliations:** College of life and Environmental Sciences, Hangzhou Normal University, Hangzhou 311121, China; 2023111010043@stu.hznu.edu.cn (J.X.); tangya678678@163.com (Y.T.); 2022210301227@stu.hznu.edu.cn (Y.J.); 2022111010007@stu.hznu.edu.cn (T.M.); zchen2603@163.com (C.Z.); 2021210315035@stu.hznu.edu.cn (J.L.); tbzm611@163.com (B.T.)

**Keywords:** *Locusta migratoria*, RNAi, fecundity, lipid metabolism, energy metabolism

## Abstract

*Fatty acid synthase* (*FAS*) is a pivotal gene in the lipid synthesis pathway and plays a crucial role in insect energy metabolism. *Locusta migratoria*, as one of the major agricultural pests, requires identification of new targets to control or reduce its reproductive capacity for effective locust pest management strategies. In this study, we focused on *L. migratoria* and identified *FAS2* as a potential target gene with functional significance in lipid metabolism and reproduction based on sequence characteristics analysis and tissue-expression patterns of five *FAS* genes. Subsequently, through RNA interference (RNAi) targeting *FAS2* expression, we assessed alterations in lipid and carbohydrate metabolism-related gene expression levels, lipid and carbohydrate contents, ovarian development, and reproductive capacity using experimental techniques such as RT-qPCR, ELISA, and morphological observations. Our findings revealed that interference with *FAS2* upregulated genes involved in lipid degradation, including *Lsd*-1, *Lsd*-2, *Lipase3*, and *Brummer*, while significantly decreasing the TAG content and fat accumulation. At the level of carbohydrate metabolism, *FAS2* silencing led to significant upregulation of key genes *TPS* and *GS* in the synthesis pathway, resulting in increased glycogen and trehalose content. In addition, *FAS2* interference resulted in a significant reduction of *Vg* mRNA expression level sand protein content in *L. migratoria*, followed by delayed ovarian development and reduced egg production. This further confirms that impaired *FAS2* function prompts *L. migratoria* to enhance lipid degradation and sugar storage to maintain the energy balance, while reducing the energy investment into reproduction. Collectively, the results of this study suggest that *FAS2* can serve as a novel molecular target for controlling *L. migratoria.*

## Simple Summary

*Locusta migratoria* is one of the most significant agricultural pests, characterized by its strong reproductive capacity and rapid reproduction rate. Consequently, identifying novel targets to control or reduce the fecundity of migratory locusts is of significant practical importance. In this study, we analyzed the sequence and tissue-expression profiles of five *FAS* genes in *L. migratoria*, ultimately screening out *FAS2* as a potential target gene due to its involvement in lipid metabolism and reproduction. Upon interference with *FAS2*, lipid catabolism was enhanced, leading to reduced lipid accumulation in both the fat body and ovaries. Furthermore, carbohydrate metabolism was altered, leading to increased glycogen storage as a compensatory mechanism to maintain the energy balance. Furthermore, silencing *FAS2* resulted in decreased lipid storage, which subsequently inhibited the expression of *Vg*, adversely affecting ovarian development and fecundity. The findings of this study suggest that *FAS2* can serve as a novel molecular target for controlling *L. migratoria*.

## 1. Introduction

Lipids serve as a vital energy source for organisms and play crucial roles in various physiological processes, including insect growth, development, immunity, and reproduction. Lipid reserves are of the utmost importance in the life history of insects [1,2,3]. Organisms generally obtain lipids from food or synthesize fatty acids de novo within cells. The biosynthesis of fatty acids involves multiple enzymes such as acetyl-CoA carboxylase (ACC), fatty acid synthase (FAS), elongation of very long-chain fatty acids protein (ELO), desaturases (FAD), and fatty acyl-CoA reductase (FAR) [4]. Initially, ACC catalyzes the conversion of acetyl-CoA to malonyl-CoA, which serves as a substrate for FAS during lipid synthesis. Long-chain fatty acids are formed through condensation with acetyl-CoA [4,5]. Free fatty acids (FFAs) undergo a series of esterification reactions to form triacylglycerols (TAGs), which are stored as lipid droplets in adipocytes. TAGs constitute over 90% of the lipids in adipocytes [6]. Accumulated TAGs are mobilized and transported as diacylglycerols (DAGs) through the blood lymph, particularly to flight muscles and ovaries, where they can either be stored or metabolized via β-oxidation pathways [7,8]. The storage accumulation and metabolic breakdown of TAGs play crucial roles in oogenesis and embryogenesis, representing approximately 30–40% of the dry weight of insect eggs and being essential for egg maturation and the maintenance of normal physiological activities [9,10].

In addition to lipids, the growth and development of organisms also necessitate carbohydrates such as glycogen, trehalose, and glucose. Trehalose, a non-reducing disaccharide that constitutes the majority of insect hemolymph sugars and serves as the primary energy source, is commonly referred to as ‘blood sugar’ in insects [11]. The synthesis of trehalose is predominantly regulated by two enzymes: trehalose-6-phosphate synthase (TPS) and trehalose 6-phosphate phosphatase (TPP), which catalyze the conversion of uridine diphosphate glucose (UDP-G) into trehalose. Subsequently, it circulates through the bloodstream to specific tissues for its functional roles [12]. Trehalose can only be utilized after being converted into glucose. Currently, only one enzyme known as trehalase (TRE) has been identified to regulate its degradation by specifically breaking down trehalose into glucose [11]. UDP-G can also be employed for glycogen synthesis, which is governed by the enzyme glycogen synthase (GS) [13]. The mobilization of glycogen is controlled by glycogen phosphorylase (GP), responsible for its breakdown into 1-phosphogluconate, then decomposed to pyruvate to release energy, or converted to glucose 6-phosphate to synthesize trehalose with UDP-G [14].

*FAS* is a highly conserved key gene in the fatty acid biosynthesis pathway, exhibiting multifunctionality with seven active sites [15]. In insects, the first reported *FAS* (also known as *FASN*) gene was isolated from *Drosophila*’s fat body [16]. Specifically expressed in the fat body, *FASN1* in *D. melanogaster* plays a crucial role, while *FASN2* and *FASN3* are predominantly expressed in oocytes; their silencing leads to lethality in *Drosophila* [17]. In *Rhodnius prolixus*, the fatty acid synthase gene *RPRC000123* exhibits high expression levels in the cuticle and plays a pivotal role in hydrocarbon precursor biosynthesis [18,19]. Moreover, during the insect diapause stage, *FAS* assumes an important function in lipid metabolism by upregulating it to facilitate lipid accumulation throughout the lipid storage phase [20]. In *Colaphellus bowringi*, downregulation of the *FAS2* in the fat body leads to diminished lipid storage and decreased stress resistance, highlighting the pivotal role of *FAS* in promoting lipid accumulation and facilitating energy storage during the diapause and late diapause stages [3]. Additionally, *FAS* is involved in regulating reproductive processes; knockdown of the *FAS* via RNA interference results in reduced fatty acid content in both ovaries and fat bodies of *Nilaparvata lugens*, accompanied by a decline in reproductive capacity [7]. For the majority of insects, egg survival relies on the utilization of previously ingested proteins, fats, carbohydrates, and other small molecules. In insects, the oocyte serves as a specialized structure responsible for the selective absorption and storage of reproductive proteins (Vg) and nutrients like TAG [21,22]. The accumulation and breakdown metabolism of TAG play a crucial role in successful egg development and embryogenesis [23]. A study revealed that upon blood feeding there was a significant upregulation in the expression of *FAS* in both the fat body and ovaries of *Aedes aegypti* mosquitoes. However, interference with *FAS* through RNAi resulted in a substantial decrease in TAG levels along with an observable reduction in egg production [24]. Lipid synthesis and storage during mosquito larval stages also hold significance for the adult lifespan, reproduction, and egg production [25].

*Locusta migratoria*, an important agricultural pest known for its robust reproductive ability and rapid reproduction speed, poses a significant threat to both the economy and ecological balance [26]. Therefore, it is of great practical significance to screen and discover new control targets that can effectively reduce or control locusts’ reproductive capacity, thereby enriching information and methods for locust pest management. RNA interference (RNAi) technology, a novel green and sustainable control strategy with high efficiency and specificity in crop pest management, has been widely employed [27,28]. In this study, we identified five *FAS* genes from the NCBI and the locust genome database. Among them, only *FAS2* exhibited high expression levels in adipose tissue. Considering that adipose tissue serves as a multifunctional organ involved in lipid storage, glycogen storage, as well as vitellogenin synthesis, we hypothesized that *FAS2* plays a role in physiological processes within adipose tissue. To validate this hypothesis, we utilized RNAi technology to silence *FAS2* expression. Our results demonstrated that silencing *FAS2* inhibited lipid accumulation significantly by causing a notable decrease in TAG content while exerting severe negative effects on reproduction. These findings offer potential research targets for future biological pest control strategies.

## 2. Materials and Methods

### 2.1. Insects for Testing

*L. migratoria* eggs were sourced from the Huaibei Locust Farm in Anhui Province, China, and incubated in containers (10 cm × 15 cm × 20 cm) with 2–3 cm of moist sand at the bottom in an artificial climate chamber at 30 ± 2 °C and 60–80% relative humidity (RH), under a L:D photoperiod of 16 h:8 h. Upon hatching, the locusts were transferred to well-ventilated insect-rearing cages (50 cm × 50 cm × 50 cm) at a density of 150–200 locusts per cage and were fed with fresh wheat shoots and wheat bran.

### 2.2. Bioinformatic Analysis

The coding sequences (CDS) of FAS1 (GenBank: MN863497.1), FAS2 (GenBank: MN863498.1), and FAS3 (GenBank: MN863499.1) were initially reported by Yang et al., [29]. FAS4 (LOCMI15800) and FAS5 (LOCMI16005) were obtained from the locust genome database (http://locustmine.org/index.html, accessed on 1 July 2022) using ORF Finder, which identified the open reading frames (ORFs) and predicted amino acid sequences of the genes. The conserved domains of the predicted FAS proteins were analyzed using SMART tool. The FAS amino acid sequences of FAS and FAS of 10 species, including *D. melanogaster* and *Bombyx mori*, were selected to build a phylogenetic tree with the bootstrap value set to 1000 [30].

### 2.3. RNA Extraction and RT-qPCR

Total RNA was extracted using the Trizol reagent (TaKaRa, Dalian, China). The RNA concentration was determined using a NanoDrop 2000 spectrophotometer (Thermo Scientific, Waltham, MA, USA). Reverse transcription (RT) reactions were carried out using the PrimeScript RT Reagent Kit (Takara, Dalian, China). The cDNA was diluted 10 times for the subsequent general polymerase chain reaction (PCR), reverse transcription quantitative PCR (RT-qPCR), and dsRNA synthesis studies.

RT-qPCR was executed using a Bio-Rad Real-Time PCR Detection System (Bio-Rad, Hercules, CA, USA). The design of all RT-PCR primers was conducted using Primer 5.0 software (Table 1). *Lmβ-actin* served as an internal reference gene. The expressions of *FAS1*, *FAS2*, *FAS3*, *FAS4*, and *FAS5* were investigated using real-time fluorescence quantitative PCR, using 10.0 μL of the PCR reaction system which contained SYBR Premix Ex Taq (5 μL; Takara, Dalian, China), forward primer (0.4 μL), reverse primer (0.4 μL), template cDNA (1 μL), and RNase-free ddH_2_O (3.2 μL). The reaction process encompassed an initial pre-denaturation at 95 °C for 3 min, followed by 40 cycles of denaturation at 95 °C for 10 s, annealing at 58 °C for 15 s, and extension at 72 °C for 30 s. The relative expression of the target gene was determined using 2^−ΔΔCT^. Three biological replicates of no less than five test individuals were established per sample.

### 2.4. Analysis of Developmental and Tissue-Specific Expression

To investigate the tissue-specific expression profile of the *FAS* gene, dissections of the head, fat body, ovary, midgut, and epidermis were performed on sexually mature female locusts. To examine the spatiotemporal expression patterns of the *FAS2* gene, fat bodies from female locusts at different developmental stages were collected, including 4th instar larvae (4L), 5th instar larvae (5L), early eclosion phase (0 h post-adult eclosion, 0 day PAE), 5 days after eclosion (5 days PAE), and 10 days after eclosion (10 days PAE). Each sample consisted of three biological replicates, each containing five locust individuals. The samples were immediately frozen in liquid nitrogen and stored at −80 °C in a refrigerator for subsequent total RNA extraction. Total RNA was extracted for RT-qPCR analysis.

### 2.5. RNAi

In order to further investigate the function of the *FAS2* gene, we employed RNA interference (RNAi) technology to downregulate the expression of the target gene, while utilizing green fluorescent protein (GFP) as a negative control. Following the manufacturer’s instructions, ds*FAS2* (482 bp) and ds*GFP* (648 bp) were synthesized in vitro using T7 RiboMAXTM Express RNAi System (Promega Corporation, Madison, WI, USA). The specific primers used for synthesizing dsRNA are listed in Table 1. Female adult locusts within 12 h after eclosion were selected for RNAi experiments, with each locust being injected with 20 μg of ds*FAS2* and an equal amount of ds*GFP* being injected into the control group. After a post-injection period of 5 days, we assessed both RNA interference efficiency and gene expression levels through RT-qPCR analysis. Fat body morphology was documented using a Canon camera, while ovary morphology was observed under a dissecting microscope (Leica EZ4 HD, Wetzlar, Germany). The total egg production of females after dsRNA injection was also recorded until their death.

### 2.6. Determination of Glycogen, Trehalose, and Glucose Content

The hemolymph supernatant collected from the samples was directly used to quantify the levels of trehalose and glucose. To determine the contents of these substances in the fat body, the weight (g)/volume (mL) = 1:10 was maintained by adding PBS to the fat body. The mixture was thoroughly ground using a grinder and ultrasonicated to ensure complete cell disruption. The resulting homogenate was then centrifuged at 2500 rpm at 4 °C for 20 min, and the resulting supernatant was used to measure glycogen, trehalose, and glucose concentrations. Each treatment included three biological replicates, with no fewer than five specimens.

The glycogen content was determined following the method described by Mollaei et al. [31], with appropriate modifications. Briefly, 30 µL of hemolymph or fat body supernatant was mixed with 600 µL anthrone solution (0.02 g anthraquinone (Sinopharm, Beijing, China) dissolved in 10 mL of 98% sulfuric acid) and reacted at 90 °C for 10 min. The light absorption at 620 nm was measured using a SpectraMax M5 (Molecular Device, Sunnyvale, CA, USA) and the glycogen content was calculated based on a standard curve.

The trehalose and glucose levels were determined as described by Wang et al. [32]. The trehalose content was determined using the anthranone method. In the first step, 30 µL supernatant was mixed with an equal volume of 1% sulfuric acid. The reaction conditions included incubation in a water bath at 90 °C for 10 min, followed by cooling at 0 °C for 3 min. In the second step, 30 µL of a 30% potassium hydroxide (Sinopharm, Beijing, China) solution was added to the previous reaction mixture under identical conditions as before. Lastly, in the third step, 600 µL of anthrone solution was added to the reaction mixture from the previous step and incubated under similar conditions as before. After completion of the reaction, the absorbance at 630 nm was measured using a SpectraMax M5 (Molecular Device, Sunnyvale, CA, USA) to calculate the trehalose content based on a standard curve. The glucose content was measured using a Glucose (GO) Assay Kit (Sigma-Aldrich, St. Louis, MI, USA).

### 2.7. Determination of Triglyceride and Free Fatty Acid Content

The haemolymph, ovaries, and fat bodies of *L. migratoria* were dissected and collected 5 days after dsRNA injection. According to the manufacturer’s instructions, the levels of TAGs and FFAs in each tissue were quantified using a TAG test kit (Nanjing Jiancheng, Nanjing, China) and a FFA content detection kit (Solarbio, Beijing, China).

### 2.8. Determination of Vg Protein Content (ELISA)

The Vg content in the fat body, ovary, and hemolymph was quantified using an indirect double-antibody sandwich ELISA method based on the protocol of Guo et al. [33] with appropriate modifications. For the hemolymph samples, the Vg content was determined from the supernatant collected after centrifugation. Tissue samples were mixed with PBS at a weight (g)-to-volume (mL) ratio of 1:10 and thoroughly homogenized using a grinder, followed by ultrasonication to ensure complete cell disruption. The resulting homogenate was then centrifuged at 8000 rpm for 10 min at 4 °C, and the obtained supernatant was used for Vg content analysis.

The specific steps were as follows: The supernatant was diluted with the coating solution (1.59 g Na_2_CO_3_ and 2.93 g NaHCO_3_ dissolved in 1000 mL sterile water) at an appropriate ratio, thoroughly mixed, and 100 mL of the mixture was added to each well of a 96-well microplate (Sangon, Shanghai, China). After sealing the plate, it was incubated at 37 °C for 4 h and then fixed. The plate was then rinsed five times with phosphate buffered saline with Tween (PBST; Sangon, China) for 1 min each time. To seal, 400 μL of a 5% skimmed milk powder solution (Sangon, China) was added to each well, followed by sealing with a film cover, incubation at 37 °C for 2 h, and washing thereafter. For primary antibody incubation, 100 μL of Vg protein antibody diluted in PBST (ratio, 1:1000) was added to each well (GenScript, Nanjing, China), after which the plate was sealed with a membrane, incubated at 37 °C for 2 h, and washed again to remove unbound primary antibody. For incubation of the secondary antibody, 100 μL of enzyme-labeled goat anti-rabbit was added to each well (diluted in PBST; ratio, 1:5000) (Biosharp, Hefei, China), after which the plate was sealed with a membrane, incubated at 37 °C for 1 h, and washed again to remove unbound secondary antibody. For the color development reaction, 100 μL TMB color development solution was added to each well (Beyotime, Haimen, China) and the plate was incubated at 37 °C for 10 min until the reaction is visible. Finally, 50 μL of 2 M H_2_SO_4_ was added to each well to stop the reaction, and the absorbance values were measured at 450 nm using a SpectraMax M5 (Molecular Device, Sunnyvale, CA, USA).

### 2.9. Data Statistics and Analysis

The Kolmogorov–Smirnov test and the Levene test were used to examine the normal distribution and homogeneity of variance of the data. The data met the assumptions of normality and homogeneity of variance. The tissue-specific expression patterns related to *FAS*s were analyzed using one-way ANOVA in SPSS 26.0 software (IBM, Armonk, NY, USA), followed by multiple comparison tests using Duncan’s method (significant differences between groups are denoted by different lowercase letters). Student’s *t*-test was employed for group comparisons, except for adipose tissue and ovarian morphological development (significance levels: “*” *p* < 0.05; “**” *p* < 0.01; “***” *p* < 0.001). Figures were generated using GraphPad Prism 9.3.1 software (La Jolla, CA, USA).

## 3. Results

### 3.1. Sequence and Phylogenetic Analyses of FASs in L. migratoria

**Figure 1:**
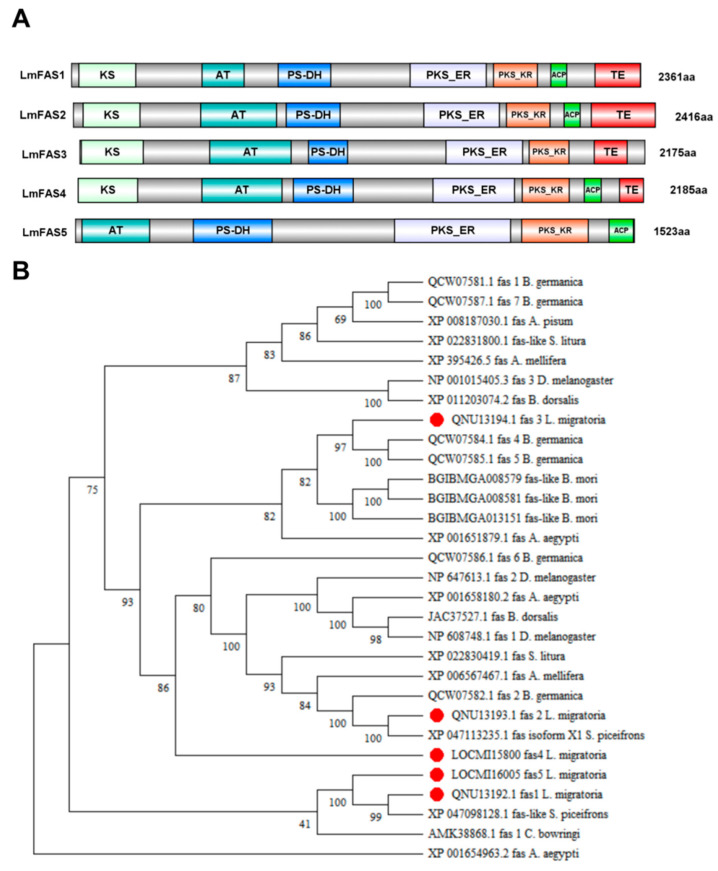
BioinformSequence and phylogenetic analyses of FASs in *L. migratoria.* (**A**) Conserved domain prediction of FAS proteins of *L. migratoria.* Animal FASs contain seven typical protein functional domains: β-ketoacyl synthase (KS), acetyl transferase (AT), β-hydroxacyl dehydratase (PS-DH), Enoyl reductase (PKS_ER), β-ketoacyl reductase (PKS_KR), acyl carrier protein (ACP), and thioesterase (TE). (**B**) Evolutionary tree analysis of locust FASs (neighbor-joining). The insect FAS protein sequences of *S. piceifrons*, *C. bowringi*, *A. aegypti*, *B. mol*, *D. melanogaster*, *A. germanica*, *A. mellifera*, *S. litura*, *B. dorsalis*, and *A. pisum* were selected to construct the evolutionary tree.

The LmFASs proteins exhibited catalytic functional regions characteristic of animal FASs, encompassing seven components: β-ketoacyl synthase (KS), malonyltransferase (AT), β-hydroxyacyl dehydrogenase (PS-DH), enoyl reductase (PKS_ER), β-ketoethyl reductase (PKS_KR), acyl carrier proteins (ACPs), and thioesterases (TEs). The FAS1, FAS2, and FAS4 protein sequences included all seven functional regions, whereas the FAS3 protein sequence lacked the ACP region, and the FAS5 protein sequence lacked the KS and TE regions (Figure 1A).

Based on the protein sequence of LmFASs, a homology search was performed using the BLAST tool available on the NCBI website. Ten insect FAS protein sequences were selected for constructing an evolutionary tree, including *Schistocerca piceifrons*, *C. bowringi*, *A. aegypti*, *B. mori*, *D. melanogaster*, *B. germanica*, *A. mellifera*, *Spodoptera litura*, *Bactrocera dorsalis*, and *Acyrthosiphon pisum*. Phylogenetic analysis revealed (Figure 1B) that LmFASs exhibit high conservation among insects. LmFAS1 and FAS5 showed the highest similarity to *S. piceifrons* FAS-like proteins, with sequence identities of 74.62% and 74.18%, respectively; they clustered together in the same branch of the phylogenetic tree. LmFAS2 and FAS4 exhibited a strong similarity to *S. piceifrons* isoform X1, with sequence identities of 94.94% and 41%. LmFAS3 displayed the closest resemblance to *B. germanica* FAS5, with a sequence identity of 47.83%.

### 3.2. Developmental and Tissue-Specific Expression of FASs in L. migratoria

**Figure 2:**
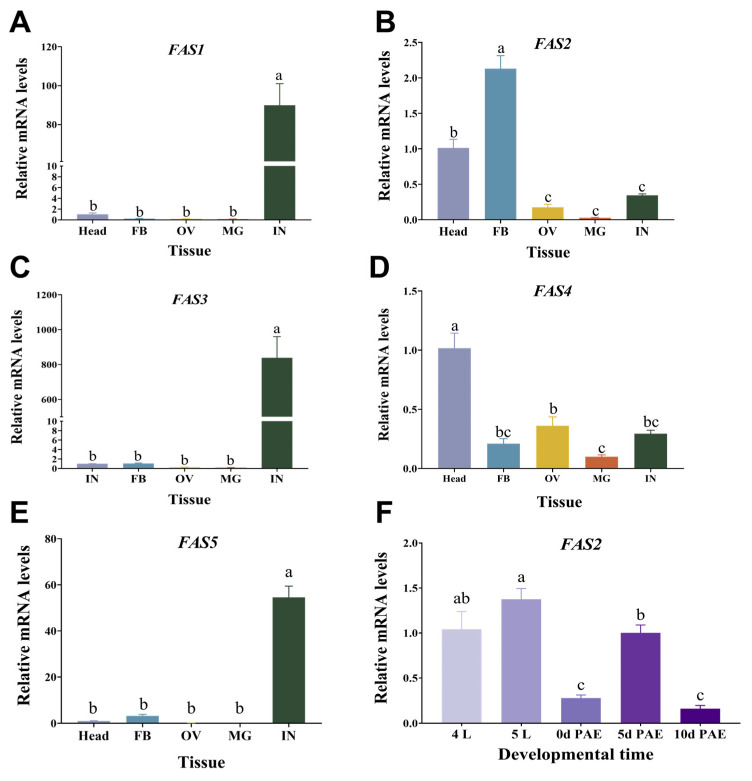
Developmental and tissue-specific expression of *FASs* in *L. migratoria.* Expression of *FASs* in the head (Head), fat body (FB), ovary (OV), midgut (MG), and integument (IN) of sexual maturation adults, including *FAS1* (**A**), *FAS2* (**B**), *FAS3* (**C**), *FAS4* (**D**), and *FAS5* (**E**). (**F**) The expression levels of *FAS2* in the fat body of locusts at different developmental stages, include the 2nd day of the 4th instar (4L) and the 5th instar (5L), early stage of eclosion (0 d PAE), the 5th day of the adult (5 d PAE), and the 10th day of the adult (10 d PAE). Values are presented as the means ± SE (n = 3). Different letters indicate significant differences among the treatments (*p* < 0.05, one-way ANOVA), and three biological replicates of no fewer than five test worms were established for each developmental stage.

The expression of *FAS*s in various tissues (head, fat body, ovaries, midgut, and integument) of sexually mature female locusts was assessed using RT-qPCR. Figure 2 presents the experimental results, revealing tissue-specific variations in *FAS* expression among locusts. *FAS1*, *FAS3*, and *FAS5* displayed high levels of expression primarily in the integument, while showing minimal expression levels in other tissues. Moreover, their expression was significantly higher in the integument compared to those observed in the head, fat body, ovaries, and midgut tissues (Figure 2A,C,E). Conversely, *FAS4* demonstrated high specificity for head tissue with significantly elevated levels compared to other tissues (Figure 2D). Notably, *FAS2* exhibited the highest expression level in the fat body, followed by the head; conversely, its expression was lowest in the midgut (Figure 2B).

Based on the tissue-specific expression, we further investigated the temporal expression pattern of *FAS*s highly expressed in the fat body. *FAS2* expression was examined in fourth instar nymphs (4L), fifth instar nymphs (5L), early eclosion (0 d PAE), 5 days after eclosion (5 d PAE), and 10 days after eclosion (10 d PAE) from the fat body. Our findings revealed that *FAS2* exhibited its highest expression levels in fifth instar nymphs, followed by fourth instar nymphs and sexually mature adults, while showing lower expression during the early eclosion stage and oviposition period (Figure 2F).

### 3.3. Evaluation of Interference Effect of dsFAS2

**Figure 3:**
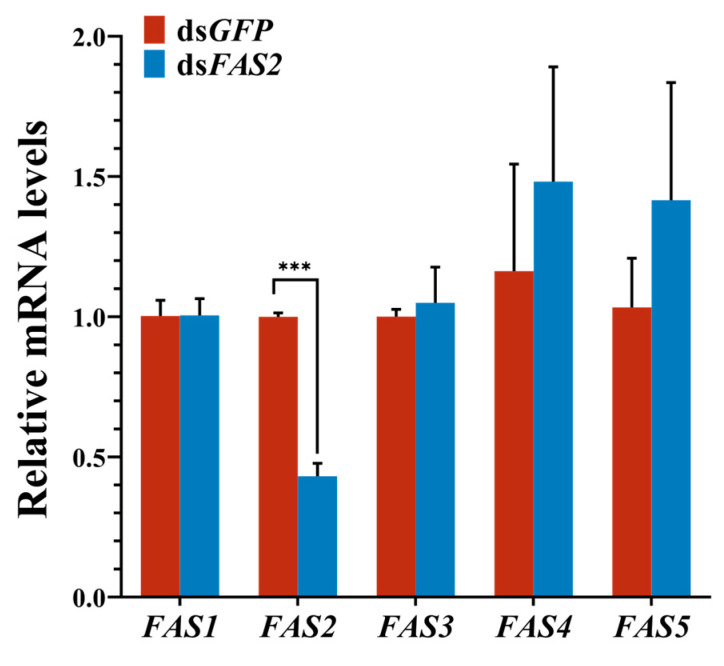
Effects of ds*FAS2* on the expression of *FASs.* The relative expression of *FAS1* (integument), *FAS2* (fat body), *FAS3* (integument), *FAS4* (head), and *FAS5* (integument) genes was examined using RT-qPCR 5 days post-ds*FAS2* injection. Values are presented as the means ± SE (*** *p* < 0.001, Student’s *t*-test). Three biological replicates of no less than five test insects were established for each treatment.

To investigate the specificity of the ds*FAS2* interference effect, the relative expression of *FAS1* (integument), *FAS2* (fat body), *FAS3* (integument), *FAS4* (head), and *FAS5* (integument) genes was examined using RT-qPCR 5 days post-ds*FAS2* injection in *L. migratoria*. The results demonstrated a significant 57% downregulation of *FAS2* expression in the ds*FAS2*-injected group compared to the control. The injection of ds*FAS2* did not show any significant influence on the expression of the remaining four *FAS* genes, which demonstrated the specificity of the interference effect of dsRNA and the obvious effect of interference (Figure 3).

### 3.4. Effects of FAS2 Silencing on Energy Metabolism in L. migratoria

**Figure 4:**
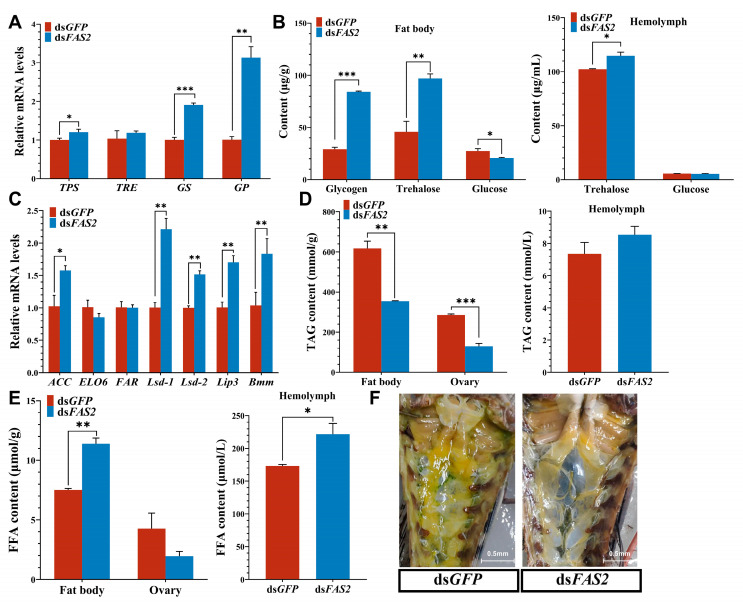
Effects of knockdown of *FAS2* gene on energy metabolism in *L. migratoria.* (**A**) Expression levels of genes associated with carbohydrate metabolic pathways were analyzed. After *FAS2* RNAi, *TPS*, *GS*, and *GP* expression were significantly upregulated. (**B**) The knockdown of effect with *FAS2* on the sugar content in fat body and hemolymph. Both glycogen and trehalose content in the fat body, as well as the trehalose content in the hemolymph, showed significant increases compared to the control group. The glucose content in the fat body was significantly reduced compared to the control group. (**C**) Expression levels of lipid accumulation-related genes after ds*FAS2* injection. The expression levels of upstream gene *ACC* and genes involved in fat decomposition, namely, *Lsd*-1, *Lsd*-2, *Lip3*, and *Bmm*, were all significantly upregulated. (**D,E**) The knockdown of effect with *FAS2* gene on the TAG and FFA content in fat body, ovary and hemolymph. The TAG content in ovary and fat body was significantly lower compared to the control group. The FFA content in hemolymph and fat body showed significant increases compared to the control group. (**F**) Photograph of the change in fat body morphology observed after *FAS2* silencing (scale bar, as shown in the Figures). Values are presented as the means ± SE (* *p* < 0.05, ** *p* < 0.01, *** *p* < 0.001, Student’s *t*-test). Three biological replicates of no less than five test insects were established for each treatment.

Silencing *FAS2* significantly upregulated the expression of key genes involved in trehalose and glycogen synthesis (*TPS* and *GS*), as well as a key gene involved in glycogen breakdown (*GP*), but had no effect on soluble *TRE* expression (Figure 4A). To explore the influence of *FAS2* on energy substance metabolism, measurements were taken for glycogen, trehalose, and glucose content after ds*FAS2* injection. After *FAS2* silencing, both glycogen and trehalose content in the fat body showed significant increases compared to the control group of 2.9 fold and 2.1 fold, respectively; similarly, there was a significant increase in blood lymphocyte trehalose content by 11.6% compared to the control group. The glucose content in the fat body, however, decreased by 27.6% following *FAS2* silencing compared to the control group (Figure 4B).

Additionally, the experimental findings demonstrated a significant upregulation of upstream gene *ACC* expression levels following *FAS2* silencing. Furthermore, the expression levels of genes involved in fat decomposition, namely, *Lsd*-1, *Lsd*-2, *Lip3*, and *Bmm*, were all markedly upregulated (Figure 4C). By quantifying TAG and FFA contents in the fat body, ovaries, and hemolymph, we observed a pronounced inhibition of TAG accumulation in both adipose tissue and ovarian tissues after the downregulation of *FAS2*. The content was significantly lower compared to the control group injected with ds*GFP*, exhibiting reductions of 42.5% and 54.4%, respectively (Figure 4D). However, upon the downregulation of *FAS2* expression, there was an increase in FFA content by 51.7% in the fat body and by 27.9% in the hemolymph compared to the control group (Figure 4E), without exerting significant effects on the TAG content in the hemolymph or the FFA content in ovaries. Moreover, morphological changes were observed within adipose tissue, where evident fat accumulation occurred within the abdominal cavity of grasshoppers belonging to the control group, while it was inhibited in the experimental group (Figure 4F).

### 3.5. Effects of FAS2 Silencing on Ovarian Development in L. migratoria

**Figure 5:**
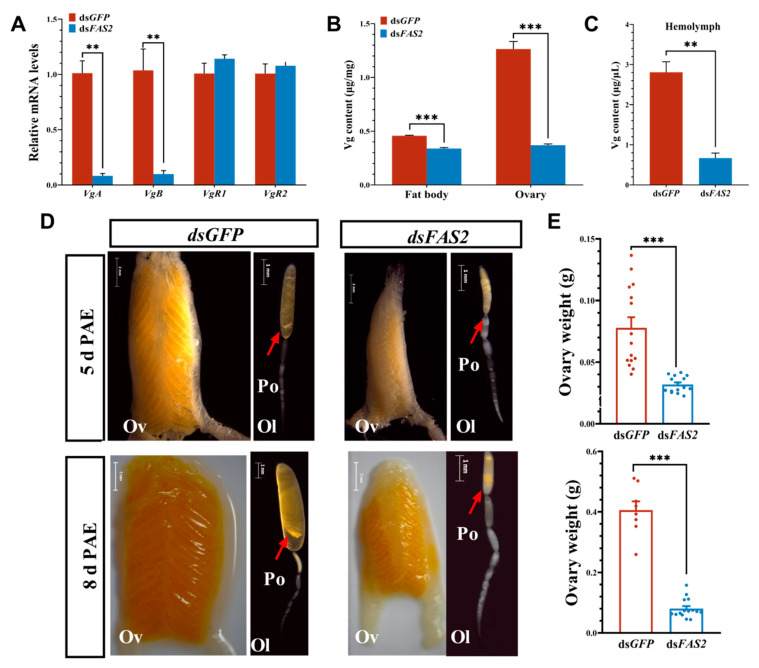
Effects of knockdown of *FAS2* gene on ovarian development in *L. migratoria*. (**A**) Effect of ds*FAS2* injection on vitellogenin gene expression. After *FAS2* RNAi, *VgA* and *VgB* expression were significantly upregulated. (**B**,**C**) Changes in Vg protein levels in fat bodies, ovaries, and hemolymph. After *FAS2* RNAi, the Vg protein levels were significantly lower compared to the control group. (**D**) Changes in ovary morphology and weights of *FAS2* low-expressing locusts. Ov, Ol, and Po represent ovary, ovarian tube, and primary oocyte, respectively. The arrows in the diagram point to the primary oocyte. (**E**) Ovarian weight was determined for each group using at least 15 dissected locusts. Values are presented as means ± SE (** *p* < 0.01, *** *p* < 0.001, Student’s *t*-test). Each treatment included three biological replicates with a minimum of five test insects. Ol morphology (scale bar = 1 mm), Ov morphology (scale bar = 2 mm), day 5 post-eclosion (scale bar as shown in the Figures).

The effects of *FAS2* silencing on ovarian development were investigated by measuring the expression of *Vgs*, *VgRs*, and *Vg* in locusts. Changes in the ovarian weight and morphology were recorded. These results demonstrate that the downregulation of *FAS2* expression hampers the expression of *Vg* and, subsequently, affects Vg synthesis. Given that locust Vg is synthesized in the fat body, transported to the ovaries via hemolymph, and selectively internalized into oocytes through endocytosis mediated by *VgR*, we investigated the mRNA expression levels of two major vitellogenin genes, *VgA* and *VgB*, in the fat body following RNAi treatment, as well as the expression levels of vitellogenin receptor genes *VgR1* and *VgR2* in the ovaries. In comparison to the control group, both mRNA expression levels and the protein content of both *VgA* and *VgB* were significantly reduced in the experimental group. Specifically, *VgA* and *VgB* expression levels decreased by 12.2 fold and 10.5 fold, respectively. (Figure 5A), while the protein content in ovaries and hemolymph decreased by 70.8% and 76.1%, respectively (Figure 5B). Morphological observations showed that a low expression of *FAS2* severely affected ovarian development. Compared to the control group, the experimental group exhibited significant atrophy in ovaries and poor development of oocytes after injection with ds*FAS2*. Ovaries in the control group appeared yellowish, with well-defined egg chambers and prominent oocytes. However, the ovaries in the experimental group appeared pale yellow, with small and inconspicuous oocytes (Figure 5C), and their weight was significantly lower than that of the control group (Figure 5D).

### 3.6. Effects of FAS2 Silencing on Fecundity in L. migratoria

**Figure 6:**
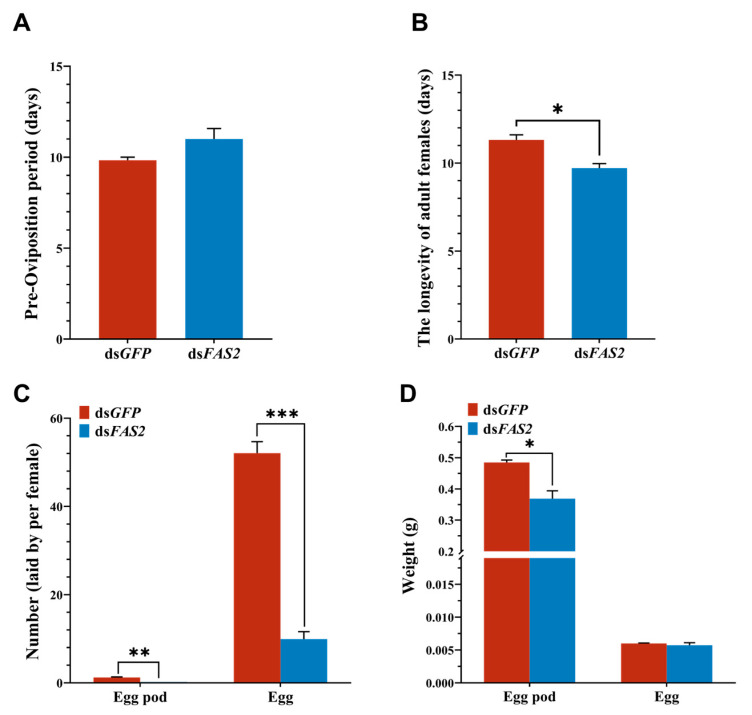
Effects of knockdown of *FAS2* gene on fecundity in *L. migratoria*. (**A**,**B**) Effect of *dsFAS2* injection on pre-oviposition and adult longevity. (**C**,**D**) Changes in number and weight of egg pods and eggs after *dsFAS2* injection. Values are presented as means ± SE (* *p* < 0.05, ** *p* < 0.01, *** *p* < 0.001, Student’s *t*-test). Each treatment included three biological replicates with a minimum of five test insects.

To further investigate the impact of *FAS2* on locusts’ reproductive capacity, we assessed indicators such as the lifespan and egg-laying ability of female locusts following dsRNA injection. Our experimental findings revealed that interference with *FAS2* expression compromised locusts’ reproductive capacity. Firstly, compared to the control group, silencing ds*FAS2* resulted in prolonged pre-spawning and significantly reduced the locust lifespan by approximately 1.6 days (Figure 6A,B). Secondly, in the experimental group, each female locust exhibited minimal egg-laying activity, with significantly lower quantities and weights of produced egg pods compared to the control group. Moreover, on average, each female locust in the control group laid 5.2 times more eggs than those in the experimental group (Figure 6C,D).

## 4. Discussion

FAS is a multifunctional enzyme composed of multiple catalytic units, which plays a crucial regulatory role in the biosynthesis of lipids in organisms [3,34]. In this study, we retrieved five FASs through the NCBI and locust genome databases and predicted the functional domains of the encoded proteins. It was found that all of them contain five functional regions including AT, PS-DH, PKS_ER, and PKS_KR. However, there are also differences among them. FAS1, FAS2, and FAS4 have complete functional unit structures with seven catalytic units containing FAS functional domains, while FAS3 lacks an ACP domain and FAS5 lacks KS and TE domains. The TE domain is usually located at the C-terminal of the FAS protein and is mainly responsible for releasing the fatty acid chain from the thioester linkage. The KS and ACP domains are also among the indispensable modules in the fatty acid synthesis pathway. Therefore, we speculate that the two cannot independently complete fatty acid synthesis. The specific mechanism awaits further study. The secondary structure composition of locust FAS proteins is similar, consisting mainly of α-helices, β-turns, extended chains, and random coils. The tertiary structures of FAS2 and FAS4 are highly similar, as shown in the phylogenetic analysis, where these two genes clustered together with a sequence similarity of 41.69%. The tissue expression results also indicated high expression levels in the head for both genes (Figure 2B), suggesting their association with head hormones or neuropeptides. FAS1 and FAS5 clustered into the same branch in the constructed evolutionary tree. The similarity of their protein tertiary structures was not high, but the similarity of their amino acid sequences was as high as 71.12%, which might be related to the post-translational modification and processing of proteins. Furthermore, it can be observed from the systematic evolutionary tree that other insects, such as *A. aegypti*, *B. germanica*, and *D. melanogaster*, possess multiple copies of FAS genes which cluster into different branches. Similarly, the five FASs in locusts cluster into different branches, suggesting their functional differentiation. To confirm this speculation, further tissue expression analysis was conducted on these five *FAS*s.

The tissue-specificity of gene expression was closely associated with their respective functions. In *R. prolixus*, the genes *RPRC002909* and *RPRC000123* encode fatty acid synthase and are primarily expressed in the integument, playing a crucial role in insect cuticular water retention [19]. The silencing of *RPRC000123* significantly reduces the content of cuticular hydrocarbons (HCs), which are essential for maintaining the internal water balance [19]. Similarly, *FAS1* exhibits high expression levels in the integument of *B. germanica* and plays a vital role in cuticular HCs synthesis, as the integrity of cuticular lipids is critical for maintaining a proper water balance within insects [35]. Expression analysis revealed that *FAS1*, *FAS3*, and *FAS5* are predominantly concentrated in the integument, with minimal expression observed in other tissues, consistent with previous findings by Yang et al. [29] indicating that *FAS1* and *FAS3* specifically function within locusts’ integument. They found that *FAS1* and *FAS3* were almost exclusively expressed in integument, and that the synthesis of an important component of HCs was significantly inhibited, indicating that *FAS1* and *FAS3* are key genes to maintain the penetration of the epidermis [36]. Therefore, we speculate that *FAS5* may also play a role in epidermal cuticle synthesis and permeability. In contrast, both *FAS2* and *FAS4* are expressed in multiple tissues, with the highest expression of *FAS2* in the fat body and relatively high expression in the head. On the other hand, *FAS4* shows high expression in the head (Figure 2B,D), which may be related to its functional location. The head is a major region for insect neuropeptide secretion, and neuropeptides play an important role in the energy balance, interacting with some molecular signals in the fat body [37]. Therefore, we hypothesize that *FAS2* and *FAS4* may influence the regulation of neuropeptides in the head. High expression levels of *FAS2* have also been found in the fat bodies of other insects such as *Rhodnius prolixus* and *Cletus bowringi* [3,19]. Furthermore, there is more evidence showing that highly expressed *FASs* play an important role in lipid accumulation within insects [34].

The insect fat body is a multifunctional organ distributed throughout the insect body, where numerous metabolic activities take place. It serves as a central hub for energy (lipid and glycogen) metabolism and plays a pivotal role in the synthesis of reproductive protein Vg [14,38,39,40]. Previous studies have consistently reported higher expression levels of genes involved in these physiological processes within the fat body [41,42,43,44]. Notably, our study observed an elevated expression level of *FAS2* on day five after locust molting (Figure 2F), coinciding with female locusts reaching sexual maturity and initiating substantial Vg synthesis [45]. Our previous research also revealed a high expression of *FAS2* in locust nymph reproductive tissues without further elucidating its function [29]. The fat body serves multiple functions, potentially leading to energy restrictions, trade-offs, and impaired function [40]. Notably, *FAS2* exhibits significant expression in the fat body of migratory locusts (Figure 2B), suggesting its crucial involvement in various physiological trade-offs within the fat body, encompassing lipid and energy metabolism, as well as reproductive protein Vg synthesis. Ma et al. [46] demonstrated that in oriental migratory locusts, the knockdown of *FAS2* following an immune challenge altered the energy allocation strategy between immune responses and reproductive processes, further indicating that *FAS2* plays a crucial role in regulating energy distribution within the fat body.

In general, excess lipids in hemolymph circulation are typically stored as TAG and can be hydrolyzed by lipases into fatty acids to provide energy during periods of high energy demand [14]. *FAS*, which plays a pivotal role in TAG synthesis, is central to lipid accumulation in insects [3,24]. The silencing of *FAS2* in *C. bowringi* significantly reduces the TAG content and inhibits lipid accumulation in the fat body [3]. In *Drosophila*, the combined mutations of *FASN1* and *FASN2* led to a significant reduction in TAG accumulation at the larval and adult stages, affecting lipid accumulation during its developmental stages [47], indicating that FAS isoforms may have compensatory effects in lipid and carbohydrate metabolic pathways, and the specific mechanism awaits further study. These findings corroborate our experimental results. In adult locusts, silencing of *FAS2* not only suppresses lipid accumulation, but also leads to a significant decrease in TAG content within both the fat body and ovaries, highlighting the critical role played by *FAS2* in lipid storage for adult locusts. Hou et al. [48] reported, through transcriptome analysis, that genes encoding fatty acid synthase enzymes such as *FAS* were markedly downregulated, while multiple lipase genes responsible for lipid degradation were upregulated in the fat bodies of locusts with mutations affecting adipokinetic hormone/corazonin-related peptide (ACP). This trend aligns with our experimental results, where expression of the lipogenesis gene *FAS2* was reduced, resulting in a significant increase in expression levels of lipolysis genes (Figure 4C). Neuropeptides, as highly diverse neuromodulators, play central roles in coordinating the energy balance, particularly in the fat body [37]. Bioactive neuropeptides generated through processing of precursor proteins are mainly expressed in central nervous system, and fine-tune distinct metabolic pathways for lipids, carbohydrates, or proteins by interacting with specific membrane receptors and downstream molecular signals in the fat body [14]. *FAS2* exhibits predominant expression in the fat body, with notable expression also observed in the head (Figure 2B). Therefore, we hypothesize that the interplay between *FAS2* and ACP further modulates lipid metabolism homeostasis in locust fat bodies. Subsequent investigations will delve into elucidating this interaction between *FAS2* and ACP. *ACC* facilitates the conversion of acetyl-CoA to propionyl-CoA, which serves as a crucial substrate for fatty acid synthesis by FAS. This condensation reaction involves acetyl-CoA and leads to the formation of long-chain fatty acids [5]. After interfering with the expression of *FAS2*, there was a significant increase in the expression level of its upstream gene *ACC*, potentially attributed to impaired FAS function resulting in elevated levels of substrate acetyl-CoA and facilitating its conversion to acetoacetyl-CoA. The release of FFA through lipid breakdown is the main way insects utilize energy, which is mediated by various lipases including *Lipase*, *Brummer*, and lipid droplet storage protein. In *Drosophila*, *Lipase3* functions similarly to lysosomes and can hydrolyze triglycerides in lipid proteins through receptor-mediated endocytosis [49]. *Brummer*, as a homolog of mammalian adipose triglyceride lipase, plays an important role in insect lipid mobilization. Activated *Brummer* interacts with the lipid droplet membrane to activate TAG decomposition metabolism, which is crucial for energy supply during fruit fly starvation periods [23]. In *Drosophila*, *Lsd*-1/*PLIN1* recruits relevant lipases such as *Brummer* to the surface of lipid droplets to promote lipid breakdown. Genes involved in lipid mobilization are essential for energy supply during starvation periods in *Drosophila* [50,51]. It is generally believed that *Lsd*-2/*PLIN2* mainly functions in lipid storage. However, recent studies have confirmed its dual role in both accumulation and breakdown of lipids [52,53]. The RNAi-mediated knockdown of *FAS2* promoted lipid mobilization in locusts. Specifically, after silencing *FAS2*, the content of FFA increased more than twofold in fat bodies and hemolymph due to significantly upregulated expression levels of genes involved in fat breakdown such as *Lsd*-1, *Lip3*, and *Bmm*. The involvement of *Lsd*-2 in lipid breakdown remains uncertain and requires further validation.

Egg production is one of the most energy-demanding events in the adult lives of female insects. In addition to Vg, large amounts of carbohydrates and lipids are required to meet the energy demands of oocyte growth [54]. GS, GP, TPS, and TRE are crucial enzymes that regulate glycogen and trehalose metabolism, with their expression levels closely associated with carbohydrate metabolism. The synthesis of trehalose is mainly regulated by TPS and TPP [12]. Current studies have shown that only TRE regulates its degradation [11]. In our study, the expression of *TPS* significantly increased, but *TRE*, which regulates the degradation of trehalose, did not change significantly, resulting in a significant increase in the content of trehalose (Figure 4A,B). The synthesis process of glycogen is regulated by GS [13]. GP degrades it into 1-phosphate glucose, and then decomposes it into pyruvate through the glycolysis process to release energy, or further converts it into 6-phosphate glucose and UDP-G to jointly synthesize trehalose [14]. Previous studies have demonstrated that the inhibition of glycogen synthesis and breakdown pathways leads to a significant reduction in trehalose levels, while glucose levels significantly increase following *GS* silencing [55]. Moreover, the downregulation of *TPS* and *TRE* in *N. lugens* results in a substantial decrease in glycogen content as part of a mechanism for converting it into trehalose to maintain adequate trehalose levels [56]. The upregulation of key genes (*TPS*, *GS* and *GP*) involved in the carbohydrate metabolism pathway leads to a noticeable increase in both glycogen and trehalose contents within fat bodies, as well as trehalose in hemolymph, while glucose content notably decreases (Figure 4A,B). Lipid metabolism is intricately linked to carbohydrate metabolism. Glycogen and fat bodies serve as crucial energy reserves in organisms. Consequently, when the accumulation of fat bodies is inhibited, migratory locusts may compensate by increasing their glycogen content to maintain adequate energy storage. During periods of fasting or when faced with high energy demands, the body mobilizes stored glycogen and trehalose as a source of energy supply [32,57]. For instance, in starved *Drosophila*, fat reserves are utilized to sustain survival by utilizing glycogen [58]. In migratory insects, coordinated carbohydrate and lipid metabolism regulate energy supply during flight. Initially, trehalose is transported to flight muscles through hemolymph circulation for providing energy. As the concentration of hemolymph trehalose decreases to a certain level, glycogen and lipids are mobilized as fuel for flight [57]. In *FAS2*-underexpressed locusts, there is a significant decrease in lipid reserves, while noticeable increases in glycogen and trehalose levels occur. Additionally, the glucose content significantly decreases. This suggests that when lipid reserves decline, glucose is converted into trehalose and stored as glycogen in fat bodies, which promotes their release into the hemolymph (Figure 4B).

For most oviparous animals, including insects, the reproductive process is an energetically demanding event, and energy reserves and mobilization play a crucial role in egg maturation [59]. During insect oogenesis, the oocyte not only assimilates Vg protein to fuel embryonic development, but also accumulates nutrients such as lipids and carbohydrates [60,61]. In *A. aegypti*, lipid storage and utilization are particularly vital for successful egg maturation, with higher energy reserves being associated with increased egg production [7,54]. Moreover, inadequate energy availability or excessive fat breakdown may exert detrimental effects on insect fertility. Under starvation stress, the depletion of energy reserves of glycogen and TAG leads to a decline in egg-laying quantity in *Helicoverpa armigera* [62], as well as an elongation of the pre-oviposition period and a shortened lifespan in fruit flies [63]. The interference with *FAS2* resulted in an extended pre-oviposition period in female locusts and a significant reduction in the adult lifespan, potentially attributed to inadequate energy allocation during reproduction due to diminished TAG reserves. Oogenesis is a prerequisite for ovary maturation and plays a pivotal role in insect reproduction by directly influencing their reproductive capacity [64]. In most insects, oogenesis is regulated by fat body production and the secretion of Vg along with other yolk protein precursors (YPPs), followed by selective uptake mediated through vitellogenin receptor-mediated endocytosis process during oocyte development [22,65]. The expression of *Vg* is modulated by lipid levels. In *N. lugens*, *LpR* impacts reproductive capacity through the regulation of lipid metabolism. RNAi-mediated silencing of *LpR* significantly diminishes the TAG content in fat bodies and ovaries, and severely impairs Vg expression, resulting in reduced egg-laying quantity [59]. Similarly, knockdown of *FAS2* substantially decreases the TAG content in fat bodies and ovaries in locusts, thereby inhibiting the synthesis, transport, and deposition processes associated with Vg protein production (Figure 5), consequently impairing or delaying ovarian development, which may contribute to the diminished egg-laying quantity and decreased reproductive capacity observed in locusts.

In summary, the interference of *FAS2* promotes lipid decomposition, leading to a reduction in lipid accumulation within the fat body and ovaries. Moreover, it modulates carbohydrate metabolism to regulate glycogen storage as an energy balance maintenance strategy. Furthermore, the downregulation of *FAS2* expression results in reduced reproductive energy allocation in locusts, thereby negatively impacting ovarian development and reproductive capacity by inhibiting Vg synthesis, transport, and absorption. Our study demonstrates the feasibility of pest control strategies targeting insect lipid metabolism and reproduction, and shows that *FAS2* can serve as a novel molecular target for the control of *L. migratoria*.

## Figures and Tables

**Table 1:** Primers for PCR.

Primer Name	F-Primer Sequence (5′–3′)	R-Primer Sequence (5′–3′)	
*FAS2*	ACGGAACAGGCACTAAA	GAACCAGGCTGATAAAG	cDNA clones
*GFP*	AAGGGCGAGGAGCTGTTCACCG	CAGCAGGACCATGTGATCGCGC	
*Actin*	GACGAAGAAGTTGCCGCTC	TCCCATTCCCACCATCACA	RT-qPCR
*FAS1*	TGTTGAAGTGCCTGGAGAT	GTGGGTTTGATGAAGGAGTTT	
*FAS2*	TTAGTGGAAAGGGAGGC	CCATACAAGGGTCAGGT	
*FAS3*	TCACTGGAACGGAAACGAAA	CCATAGCAAATGCAAAGGGT	
*FAS4*	ATCGCACTATCAGGAC	CTACTATGAAAGGCAAC	
*FAS5*	CCACCAGTTGTGATGAG	AACAGAAACCCGCAGA	
*ACC*	GTGTGTTGGAGCCAGAAGGAAT	CACTTGGAAGGTTAGGAGAGGA	
*ELO6*	CTGCAATGACTCTGGTCCGATAA	GCGCTGGTCACTCCTGTTGTC	
*FAR*	CACGGCGTACTGTCACTTG	TCAGCACTGGTAAACCCTTC	
*Lsd*-1	TGTCACTTGGAGGAGAAAA	AAGGTCGGAGTATCAGCAC	
*Lsd*-2	GCTCCGAAAATGGAATGC	TGCCTCAGCCGTTGATAGT	
*Lip3*	GGTCGGATTTGATGCC	TGAGCCAGGGTCTTTGTA	
*Bmm*	ATCACTGACGAGGGTCTACGA	ATACTGGTGTTGGCGAGGTT	
*TPS*	AGACGAACGGACACTACGAATGA	ATCCTCCCTTAGCGAACCCATC	
*TRE*	GCACTCCATAATCAAGCAGCAC	TAATGAACCATCGCCCAGAG	
*GS*	ACTCCGAATGGTCTCAATGTCA	GGTAGGGAATATCAGGAATGCA	
*GP*	CCCTGGTGACCTAGACAAACT	GGGTGTCATCTCATAGAAATCG	
*VgA*	CTCTTTCGTCCAACAGCCG	CTCGCAACCATTCCCTTCA	
*VgB*	GGCAGTTTTGCTTATTATGGG	TTCCGGGTTTGACAGTTGG	
*VgR1*	ATAAAGGTCTACCATCCAGCCC	GACAGGCACAGGTGTAGGAGTT	
*VgR2*	GGCAAAAGGGATCACTCGA	GCCACCATCAGCCCAAAAT	
ds*GFP*	TAATACGACTCACTATAGGGAAGGGCGAGGAGCTGTTCACCG	TAATACGACTCACTATAGGGCAGCAGGACCATGTGATCGCGC	dsRNA synthesis
ds*FAS2*	TAATACGACTCACTATAGGGACGGAACAGGCACTAAA	TAATACGACTCACTATAGGGGAACCAGGCTGATAAAG	

## Data Availability

The dataset used is available on request from the authors. The raw data supporting the conclusions of this article will be made available by the authors on request.
